# Low-Cost Microplate Reader with 3D Printed Parts for under 500 USD

**DOI:** 10.3390/s22093242

**Published:** 2022-04-23

**Authors:** Jonathan E. Thompson

**Affiliations:** School of Veterinary Medicine, Texas Tech University, 7671 Evans Dr., Amarillo, TX 79106, USA; jon.thompson@ttu.edu

**Keywords:** 3D printing, additive manufacturing, 96-well plate, low-cost science, open access, open source

## Abstract

A 96-well microplate reader for absorption spectroscopy was designed, constructed, and tested at a total cost of ca. 500 USD. The reduced cost of the device represents the major technical contribution of this manuscript, as costs were reduced 7 fold from previous reports. The device was able to achieve 3σ limits of detection of ca. 0.01 absorbance units (AU) over a 60 second measurement for the mid-visible wavelength range. Component parts are either commercially available, or 3D printed from plans. Analysis wavelength can be altered throughout the visible region through use of various photographic or theatrical filters. This feature allows the well plate reader to be used for typical laboratory assays such as cell population estimation by optical density (OD) at 600 nm, or enzyme-linked immunosorbent assays (ELISA) at 450 nm. This manuscript reports on the motivation and process of constructing the device, lists required parts, presents data demonstrating device function, and provides the community of scholars with plans to reproduce the work. The device can be reproduced in laboratories lacking sufficient resources to purchase commercially available options and this outcome contributes towards empowerment of individuals and equity of scientific enquiry.

## 1. Introduction

Open-access science aims to increase transparency, expand access, and broaden the range of research activities [1,2,3]. Central to this effort is the need to preserve and even enhance the ability to conduct scientific enquiry. Access to enquiry should be conserved across national and social boundaries, and investigators with vastly varying resources empowered to conduct high-impact science. Towards this goal, techniques of additive manufacturing (3D printing) and open-source science have been emerging to support quality science at low cost [4,5,6,7,8,9,10]. Inexpensive sensing has empowered study of the environment, biotechnology, and education [11,12,13,14,15,16,17]. Specifically, open-source fluorescence imagers [18,19], spectrophotometers [20], turbidostats [21], and robotic pipettetors [22,23] have been developed and described in the literature.

One staple device present in modern laboratories is the 96-well plate reader. The 96-well format microplate reader is used for a variety of applications including enzyme-linked immunosorbent assays (ELISAs), protein quantification assays, and turbidity-based cell population assays. Considering the ubiquity of the microplate format, several publications have appeared in recent literature which describe attempts to create low-cost alternatives. Several investigators have reported smart phone-based designs for point-of-care analysis [24,25,26]. Fu et al. [24] constructed a reader which relies on a smart phone’s optical sensor to measure transmitted light through liquid samples. To prove the validity of the device, smart-phone-based microplate readers were applied to detect several analytes through spectrophotometry. Similarly, Chen et al. [25] developed an innovative smartphone colorimetric reader that integrates the ambient light sensor with a 3D printed attachment for the readout of liquid colorimetric assays. Berg et al. [26] 3D printed an opto-mechanical attachment to hold and illuminate a 96-well plate using a light-emitting-diode (LED) array. LED light was transmitted through each well, before being collected into 96 individual optical fibers. Captured images of this fiber bundle were acquired with a smart phone and processed for light absorption. Using the system, the authors successfully tested the mobile platform using ELISA tests for mumps, measles, and herpes virus with >98% accuracy.

An open-source design for a more traditional microplate reader has also been described previously. In 2019, Szymula et al. published a paper outlining the construction and performance of an open-source plate reader [27]. The extensive Supplementary Materials provided and accompanying online materials outlines everything needed to fabricate and use the plate reader at a cost of slightly under 3400 USD. The work of Szymula et al. is comprehensive and yields a complete solution; however, the 3400 USD cost may not allow inclusivity for all laboratories. In addition, portability and minimization of cost are crucial for use in small production facilities or in remote veterinary medicine practices taking place on farms, dairies, or ranches. Given my interest in open-access science, analytical chemistry, and electronics, I was motivated to design and construct an absorbance-based plate reader at even further cost reduction. This manuscript serves as the deliverable of this effort.

## 2. Materials and Methods

### 2.1. Construction of Plate Reader

**Figure 1:**
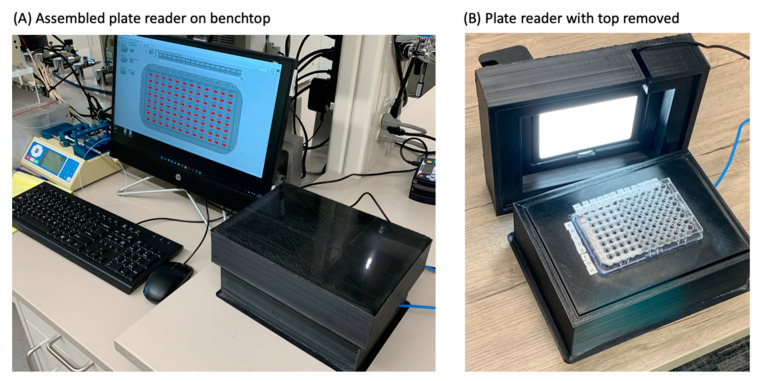
(**A**) Plate reader on lab bench prior to use. (**B**) Plate reader with top removed and no color filter.

The plate reader was constructed from a combination of 3D printed and commercially sourced components. Section 2.1.1, Section 2.1.2, Section 2.1.3 and Section 2.1.4. outline the device’s construction and Figure 1 depicts the apparatus below.

Table 1 lists parts, sources, and approximate costs for items required for construction. In final form, the plate reader measures 25 cm × 19 cm × 13 cm, has a mass of approx. 1.1 kg, and operates on USB power.

#### 2.1.1. Overview of Plate Reader Light Detection Electronics

Absorbance detection was achieved on 96 individually addressable channels through use of individual photodiodes (Digikey 1830-INL-5APD80-ND). Photos and circuit diagrams are provided in supplementary information. A custom part (Photodiode Mount) was designed, and 3D printed to hold the 96 photodiodes in a spatial configuration which matched the dimensions of a traditional 8 × 12 well plate. Photodiodes were inserted into holes of the printed part and then glued in place using epoxy with the anode and cathode extending from the bottom surface of the mount. Photodiode anodes were bent and soldered together and grounded at the data acquisition board (NI USB-6009). Each of the 96 individual cathodes were soldered to a short jumper wire and routed to a dedicated channel of a 16-channel multiplexer board (DigiKey 1568-1181-ND). Six multiplexer boards are required for the plate reader. Each multiplexer board has 16 input channels labelled C0 through C15 which are wired directly to photodiode cathodes. The multiplexers are critical components of the plate reader because they function like analog rotary switches. Only one of the input signals is routed to the signal out of the multiplexer board at a time. A 4 bit control signal presented to multiplexer board channels S0 → S3 sets which photodiode channel is being probed at a given point in time. By sequentially adjusting the binary input value set to the S0 → S3 lines from 0 to 15, each of the 16 photodiodes can be individually addressed over time. This process is repeated in parallel for all 6 of the multiplexer boards, which produces six analog signals from photodiodes at a particular point in time. A National Instruments data acquisition board (NI USB 6009) was used to measure the analog voltage signals on the six individual channels. The voltages were logged to a PC using NI LabView software thru code written in house. The digital output lines P.O.0 through P.O.3 were used to assign a binary value to the multiplexers in an automated fashion using the source code.

A data acquisition sequence begins by assigning binary zero (“0,0,0,0”) to the four digital output pins of the USB-6009 board and all multiplexer inputs wired to them. Analog signals are then collected serially from the six multiplexer boards via channels AI0 through AI5 of the USB 6009 and voltage values stored in a numeric array in the software. Next, binary counter value is incremented and written to the digital output pins and the analog signal collection continues with values appended to the array. This sequence continues until binary values up to 15 are written and data collected. A time delay of 10 milliseconds is used after switching binary values to assure sufficient time is provided to acquire representative signal. This assures all 96 wells are probed individually through an ordered and consistent sequence. In words, this process seems complicated, but the computer can execute the required commands to scan all 96 wells within a few seconds.

#### 2.1.2. 3D Printing

Several components to the plate reader were 3D printed using black acrylonitrile butadiene styrene (ABS) print filament material at the Texas Tech University MakerSpace facility using a PolyPrinter model 229. Parts were designed at tinkercad.com by the author and exported as STL files before being submitted for printing via electronic mail. The source STL files have been included as supplementary information as part of this manuscript. All files can be downloaded at ThinkTech (https://ttu-ir.tdl.org/handle/2346/88836 (accessed on 25 February 2022)), the online repository for research data for Texas Tech University.

#### 2.1.3. Description of Software

**Figure 2:**
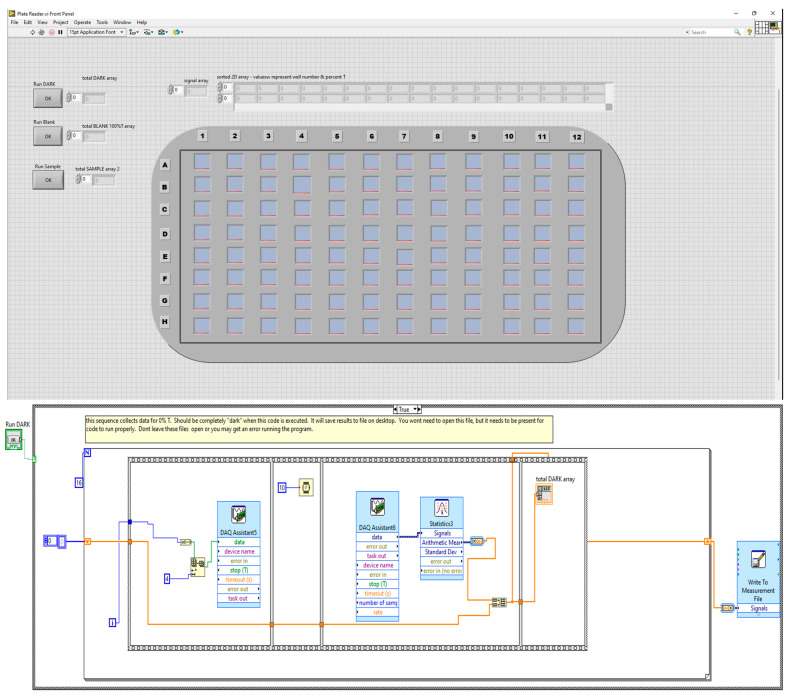
Portion of plate reader code written in LabView. (**Top**) Front panel user interface. (**Bottom**) Block diagram.

The software used to control the plate reader and collect data was written in National Instruments LabView 2017. The code’s architecture was designed for continuous run mode of operation. Three Boolean case structures were placed on the block diagram to execute upon the user clicking a certain button. The first case structure executes code when the user collects a dark spectrum. This should be carried out when all light is blocked from the photodiode array (corresponds to 0% transmittance). Figure 2 below illustrates this code.

When the user clicks front panel button, this loop executes to collect dark signals. For loop iterates 16 times, carrying out a sequence in which a 4 bit binary number is written to digital output channels for control of the multiplexer boards, followed by 10 ms delay, followed by acquisition of analog signals from photodiodes and storage to the dark signal array. After the sequence structure completes, the loop counter increments and additional channels of the multiplexer are probed. The signals are then stored to a file on the desktop of the PC. Very similar case structures also exist for the 100%T signals and sample. The code for the ‘Sample’ also includes means to compute percent transmittance (%T) from all data collected and also updates the front panel visual indicator. The LabView VI to control the plate reader and obtain data is available for download at ThinkTech (https://ttu-ir.tdl.org/handle/2346/88836 (accessed on 25 February 2022)).

#### 2.1.4. Light Source

The source of light was an array of 66 LEDs placed behind a diffuser (Neewer Dimmable 5600K USB LED Video Light, Amazon). The lighted panel measures roughly 12 × 8 cm, a size compatible with conventional 96-well plates. The plate reader top is designed with a cut-out region to precisely fit the light panel and its USB cable that provides power. The light panel was affixed to the plate reader top using small piece of Velcro. The plate reader top can then be inverted and placed on the bottom of the plate reader for use. While LEDs are known to have documented advantages as spectroscopic sources [28] (low 1/*f* noise and conversion efficiency), it was noted that the LED panel generates significant heat, which could affect kinetics experiments or particularly labile samples. It is recommended the LED panel is switched off when not in active use.

**Figure 3:**
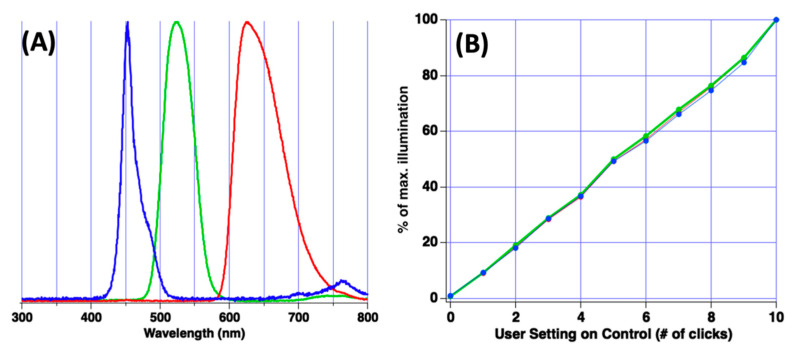
(**A**) Plot of illumination spectra from the LED panel when using the blue, green and red filter. (**B**) Plot of fraction of maximal illumination vs. intensity setting set by end-user.

Strong emission of light by the source with wavelengths between 450–650 nm was noted, and use of the microplate reader throughout this spectral region is possible. However, selection of monochromatic light is required for absorption spectroscopy. Theatrical filters obtained from Lee Filters were used for spectral selection. For this work, primary red (Lee filter 106), primary green (Lee filter 139), and dark blue (Lee filter 119) filters were chosen. Figure 3A reports the resulting illumination wavelengths incident upon the plate when these three filters were used in conjunction with the source. Peak illumination through the blue filter was observed at 450 nm, the green filter was 530 nm, and the red filter was 630 nm. None of the filters were particularly high performing as spectral width at base of peak was approx. 100 nm. However, the filters do provide an inexpensive, general-purpose solution.

The LED panel is equipped with a simple to use control with on/off feature and light intensity adjustment. In product literature, the manufacturer of the LED panel suggests that the intensity control increases the output power of the light panel by 10% each manual click of the control. If confirmed, this feature of the LED panel is quite convenient for the analyst as it can be used to assess linearity and accuracy of percent transmittance measurements (%T) for each microplate well. To confirm the manufacturer claim, an Ocean Optics Jaz bench-top spectrometer was used to measure LED panel intensity as the intensity control was varied. Figure 3B reports results of this analysis at wavelengths of 450, 530 and 630 nm (blue, green, red, respectively).

It was observed that the user control did exhibit linear control over illumination intensity for all three wavelengths. However, the exact percent increase or decrease per click was wavelength dependent and not necessarily 10%. While not tremendously precise, measurement results suggest the illumination intensity is altered linearly per user click of the control.

## 3. Results

### 3.1. Detector Linearity

**Figure 4:**
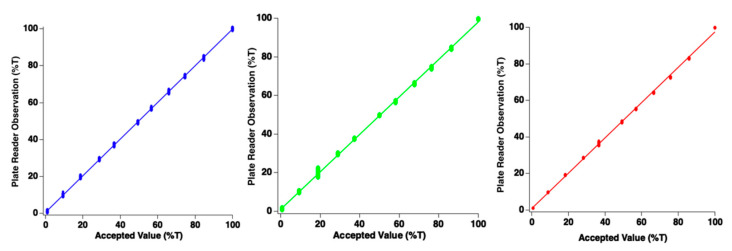
Plots of plate reader photodiode linear response for blue, green, and red channels. Each data point represents a single channel/sensor. It should be noted that ellipses viewed are each clusters of 96 individual data points. Accepted value of illumination intensity was obtained with commercially available spectrometer.

The photodiode detectors must produce signals which are linearly proportional to incident light power. To assess whether this condition was met, an experiment was conducted in which the end-user manually adjusts the incident illumination using the control. Since the accepted value for illumination intensity was measured in a separate experiment (w/Ocean Optics Jaz spectrometer), results could be directly compared at the red, green, and blue wavelengths. Figure 4 reports data which assess linearity of response. Data points in this figure represent individual measurements from a single well of the microplate for each intensity setting of the LED light panel. Note data points overlap in clusters because the 96 points form a small cluster of data around the measurement. For all spectral channels, linearity was exceptional, indicating that the plate reader performs as designed. For the red wavelength, the slope of the best-fit line was 0.97 with an intercept of 0.61%. The blue channel demonstrated a slope of 0.99 with intercept of 0.73%. The green channel demonstrated a slope of 0.97 with an intercept of 1.03%. Since all slopes were very near unity, good agreement between photodiode measurements from the plate reader and the commercial device has been observed.

### 3.2. Demonstration of Device Utility

**Figure 5:**
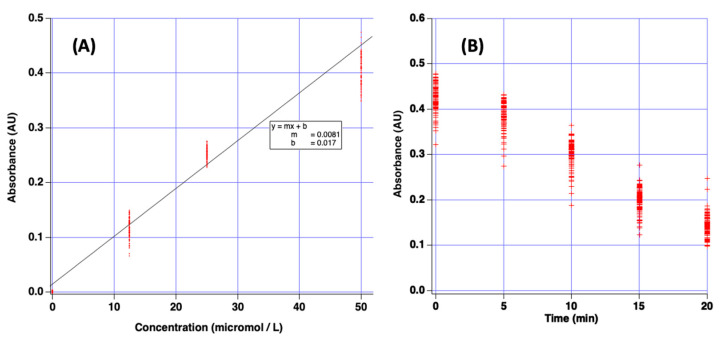
(**A**) Beer’s law plot of absorbance vs. molarity for several concentrations of crystal violet dye as measured with the plate reader and green filter. (**B**) Plot of measured absorbance vs. time for bleaching reaction of crystal violet dye with sodium hydroxide. For both plots, a single data point represents one measurement from a single well of the microplate.

A Beer’s law plot was prepared by measuring the absorbance for solutions of varying concentration of crystal violet dye using the plate reader. The resulting data are reported in Figure 5A. The equation of best-fit line was y = 0.0081x + 0.017 with R^2^ = 0.97. This plot illustrates 384 individual data points, each point is the observed measurement for a single well on the plate for a specific concentration of crystal violet. These data are crucial because they confirm the linear relationship between absorbance and crystal violet concentration. Since this is a user-built device, it could be subject to non-ideal behavior due to poor design or other unknown reasons. Linear Beer’s law plots demonstrate the device’s utility for the intended purpose.

To further demonstrate the utility of the device, a simulated kinetics experiment was conducted to measure the bleaching of crystal violet dye. It is known that a solution of crystal violet (purple dye) will lose its color upon reaction with a solution of hydroxide ion [29]. This chemical reaction provides an opportunity to report a simple demonstration of the plate reader’s function because absorbance is expected to change over time. The green spectral filter produces wavelengths which match the absorption spectrum of crystal violet.

In this experiment, 100 μL of 0.01 M sodium hydroxide solution was added to each of the 96 wells and used as spectroscopic blank to set 100% T. Then, 100 μL of 100 μM crystal violet solution was added to each well and the %T for each well noted with the plate reader at 5 min intervals. Resulting data are presented in Figure 5B. Indeed, the indicated absorbance values fall, indicating the bleaching reaction proceeded over the 20 min experiment. These experiments demonstrate that the low-cost plate reader can be used for simulated laboratory experiments.

## 4. Discussion

A low-cost plate reader has been designed and demonstrated which reduces the cost by approx. 6 fold from the previous work of Szymula [27]. This device can be used to perform routine analysis in low-resource laboratory environments. While successful, the device can be improved in the future. First, the current operating software is written in LabView (National Instruments) and provided as supplementary information. One caveat of the current study is that the end-user must have access to this software package. While LabView is commonly used in research laboratories, professional versions of LabView may cost several thousand dollars. However, National Instruments now makes LabView Community Edition available for download free of charge for non-commercial use (www.ni.com, accessed 9 March 2022). Regardless, an open-source, stand-alone executable data acquisition program would simplify the microplate reader system. Second, the LED light panel array matches the size of the microplate well. However, it does generate significant heat and can drift in intensity over time. Future versions of the microplate reader may incorporate engineering controls to account for this. This could include active ventilation of the plater reader or use of heat sinks. In addition, designated cells of the 96-well plate could be used for referencing light intensity or setting the blank during experiments in a similar fashion to dual-beam spectrophotometer designs.

## 5. Conclusions

A low-cost plate reader for 96-well plates has been designed, constructed, and tested. The plate reader hardware can be built for less than 500 USD, and plans are provided as a contribution of this work. The plate reader successfully performed laboratory experiments simulating common protocols (kinetics experiments and optical density experiments). The plate reader design may enable research within low-resource environments.

## Figures and Tables

**Table 1:** Parts list and approximate cost in US Dollars for plate reader hardware.

Item	Source	Cost (USD)
Photodiode Mount	3D printing	$2.04 ^1^
Top for Plate Reader	3D Printing	$37.47 ^1^
Collimation Screen	3D Printing	$2.04 ^1^
Base for Plate Reader	3D Printing	$40.25 ^1^
Data Acquisition Card	National Instruments USB-6009	$239.99 ^2^
Misc. Wire and Solder	various	$10
Light Source	Neewar Dimmable 5600K USB LED	$32.39 ^3^
Colored Filters	Lee Filters	$43.30 ^4^
Qty 6 16-Channel Multiplexer	Sparkfun CD74HC4067	$33
96 Photodiodes	Digikey 1830-INL-5ANPD80-ND	$17.29
TOTAL HARDWARE COST	-	$457.77

^1^ Parts printed at Texas Tech University Maker Space. Prices based on materials/consumables plus small user fee. Exact print costs may vary. All plans for 3D printing can be downloaded at ThinkTech (thinktech.lib.ttu.edu (accessed on 25 February 2022)), Texas Tech University’s online repository for research data, accessed 25 February 2022. ^2^ See Amazon.com (accessed on 9 February 2020) USB Data Acquisition Card Module 779026-01 DAQ for National Instruments NI USB-6009, accessed 9 February 2022. ^3^ See Amazon.com (accessed on 9 February 2022) Neewar Dimmable 5600K USB LED, accessed 9 February 2022. ^4^ See Amazon.com (accessed on 9 February 2020) Lee Filters Color Effects Pack, 12 Sheet Pack of Pre-cut 12 × 12” Lighting, accessed 9 February 2022.

## Data Availability

Limited data, software, and 3D print plans can be downloaded at ThinkTech (https://ttu-ir.tdl.org/handle/2346/88836 (accessed on 25 February 2022)), Texas Tech University’s online repository for research data.

## Supplementary Materials

The following supporting information can be downloaded at: https://www.mdpi.com/article/10.3390/s22093242/s1, User Guide: platereaderwiring.pdf; Files: 3D Printing Plans (Collimation screen.STL; base for plate reader-2.STL; microplate reader top.STL; photo diode mount. STL; Software: Plate Reader.VI.
