# Supplementary material for: Low-Cost Microplate Reader with 3D Printed Parts for under 500 USD
Source: Sensors (Basel). 2022 Apr 23;22(9):3242. doi: 10.3390/s22093242 (PMC9103534; doi:10.3390/s22093242)

Photodiode mount (photo diode mount.stl)

Reverse side of photodiode mount. Illustrating individual photodiodes (red arrows) epoxied into alignment holes and wiring. It is unlikely that a reproduction of this work will wire the photodiodes to multiplexer in exactly the same order. You will need to find the order for your device and update the order list within the array constant in the software VI. The array has 96 elements corresponding to A1, A2, A3… thru H12. You can easily find which photodiode by making a mask which blocks all light except for a ¼” hole. When hole is placed above a photodiode, a signal can be observed in the signal array.

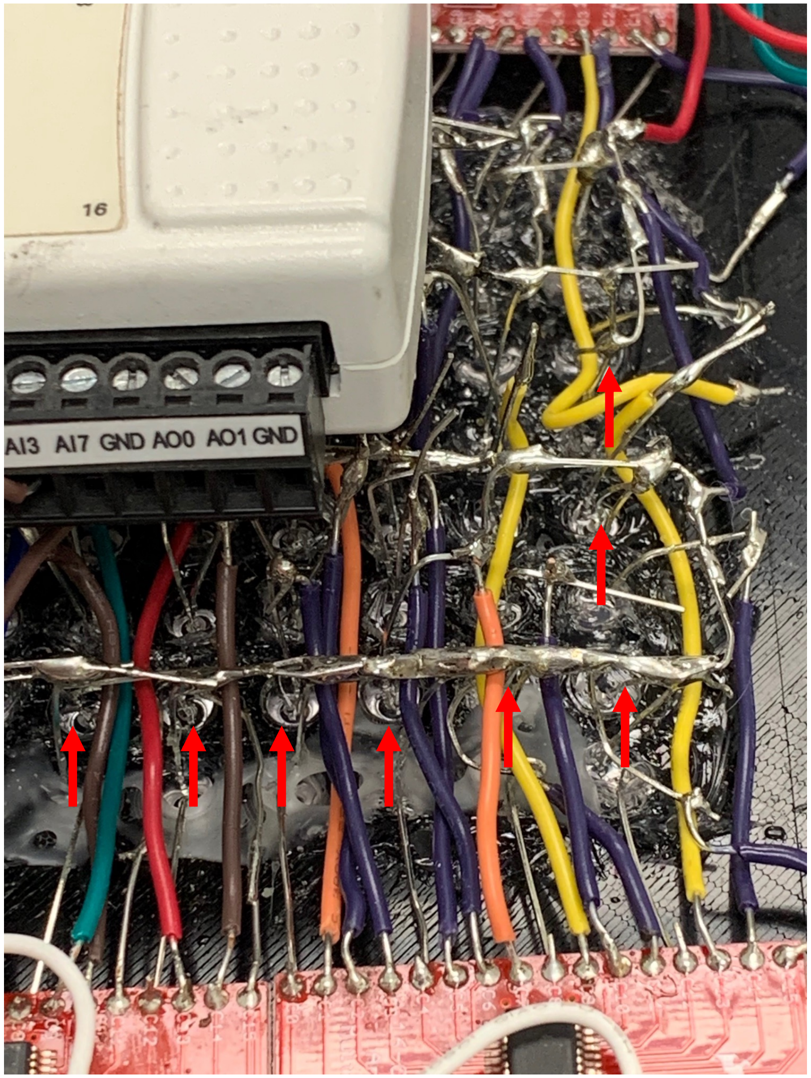

Top surface of photo diode mount – well plate sits in rectangle for alignment

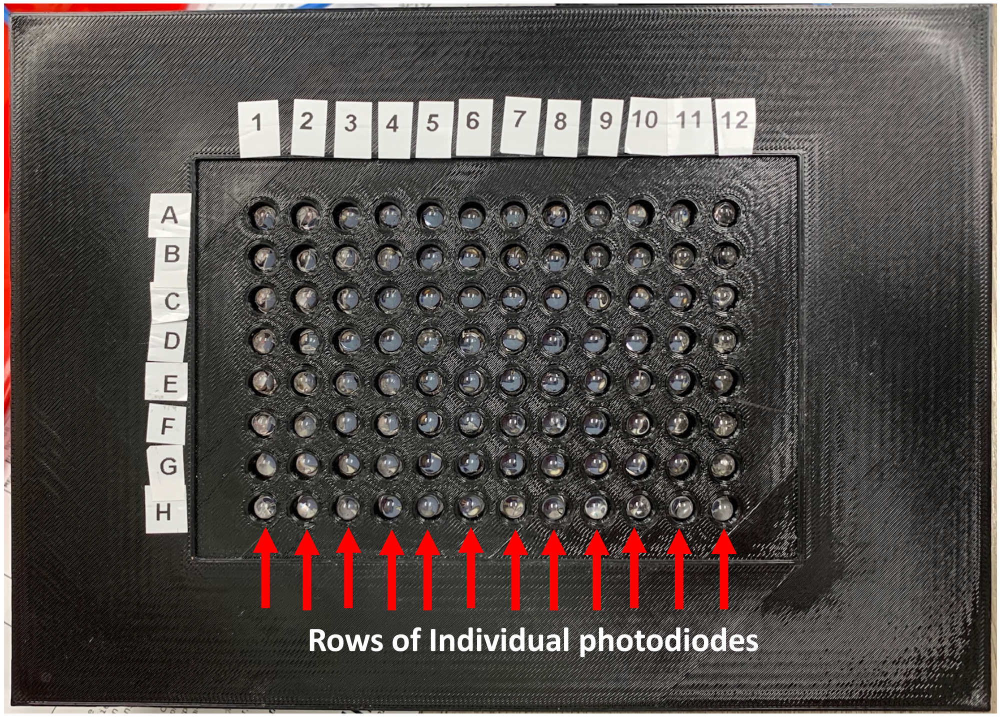

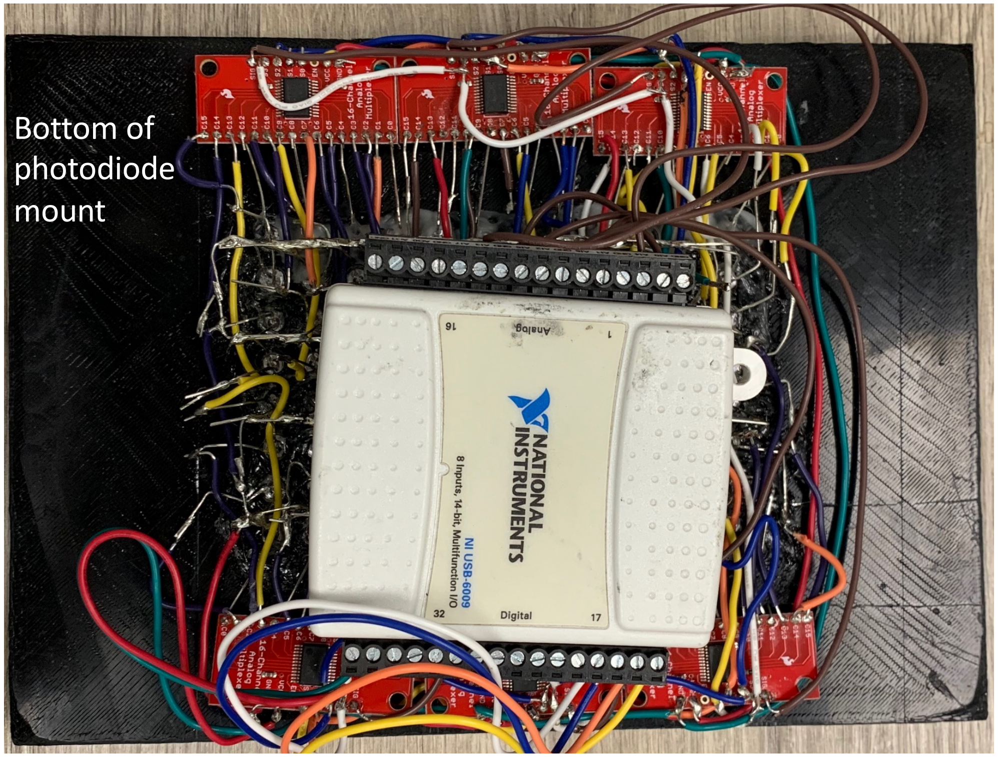

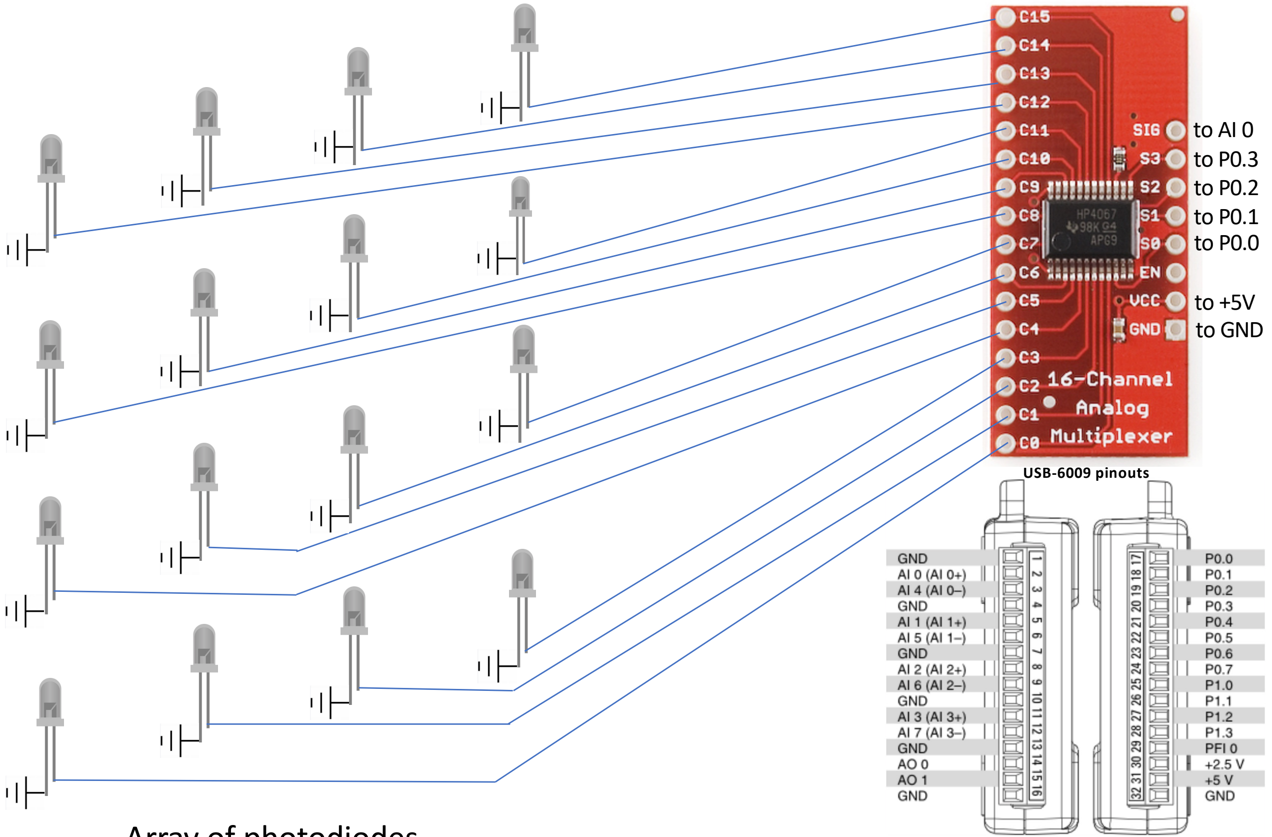

This diagram depicts 1/6 of the entire device circuit (only 1 of 6 multiplexer boards). All multiplexers will be wired the same for S0 thru S3. However, SIG lines will be routed to AI1 thru AI5 for the 5 additional multiplexer boards. All boards must be wired to +5V and GND. EN has no connection.

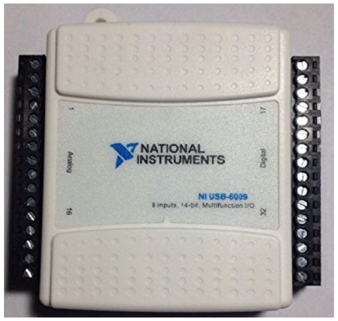

Array of photodiodes
(only 16 shown; remaining 80 will be wired to additional multiplexer boards)

In software, initialize for loop count to 0 and write this value to 4-bit counter on digital out lines

Wait 10 millisecond delay

acquire analog input data from each of the six multiplexer boards serially on six channels of DAQ board. Acquire 200 datum for each channel at 5kHz and average result. Store results for all six channels to data array.

for loop count increments +1 and new value is written to 4-bit counter on digital out lines. This causes all six multiplexer chips to switch channels

After N = 16 loop iterations

Loop ends and array results are stored to file on computer's desktop. Three files may be present for Dark signals, Blank or 100% transmittance, and one file for the sample

Plate Reader Cover (micro plate reader top.stl)

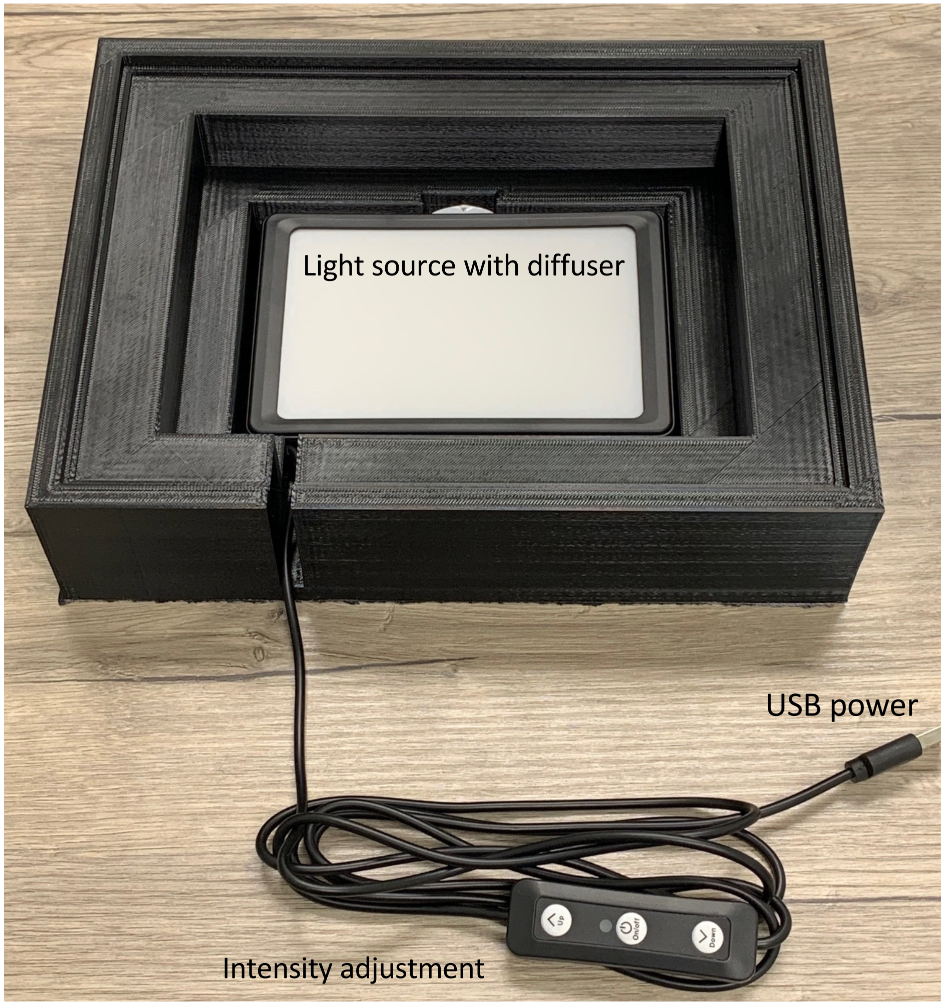

Top surface of plate reader. USB cable exits thru small slit.

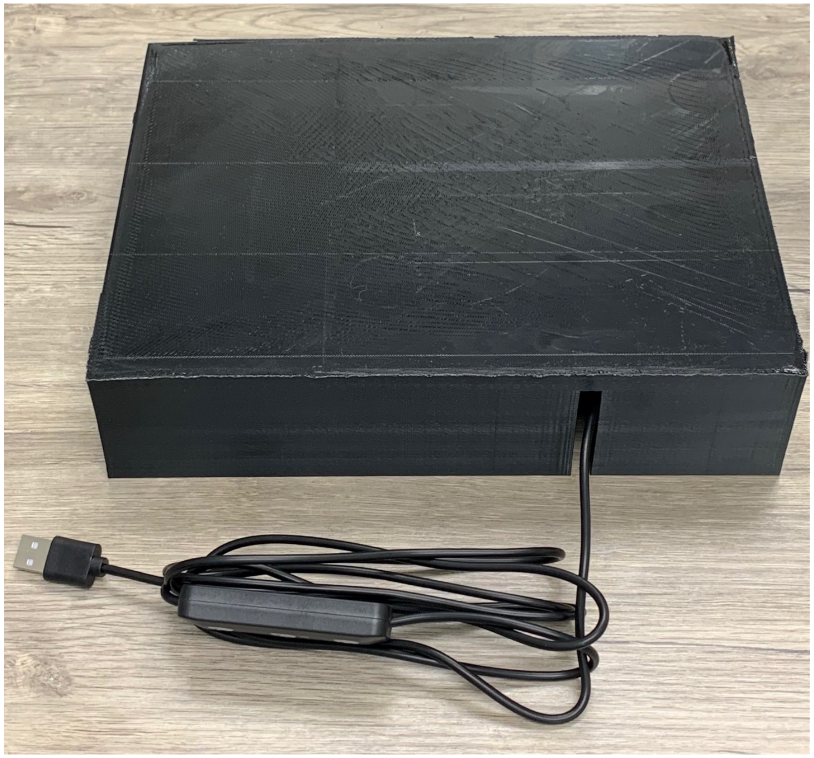

Light source turned on. LED array is similar in size to well plate

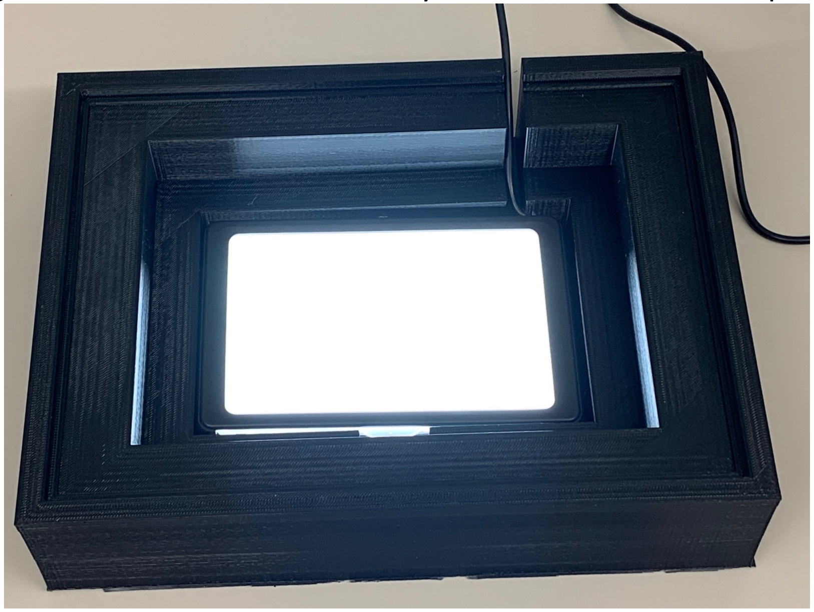

Plate Reader Base (base for plate reader-2.stl)

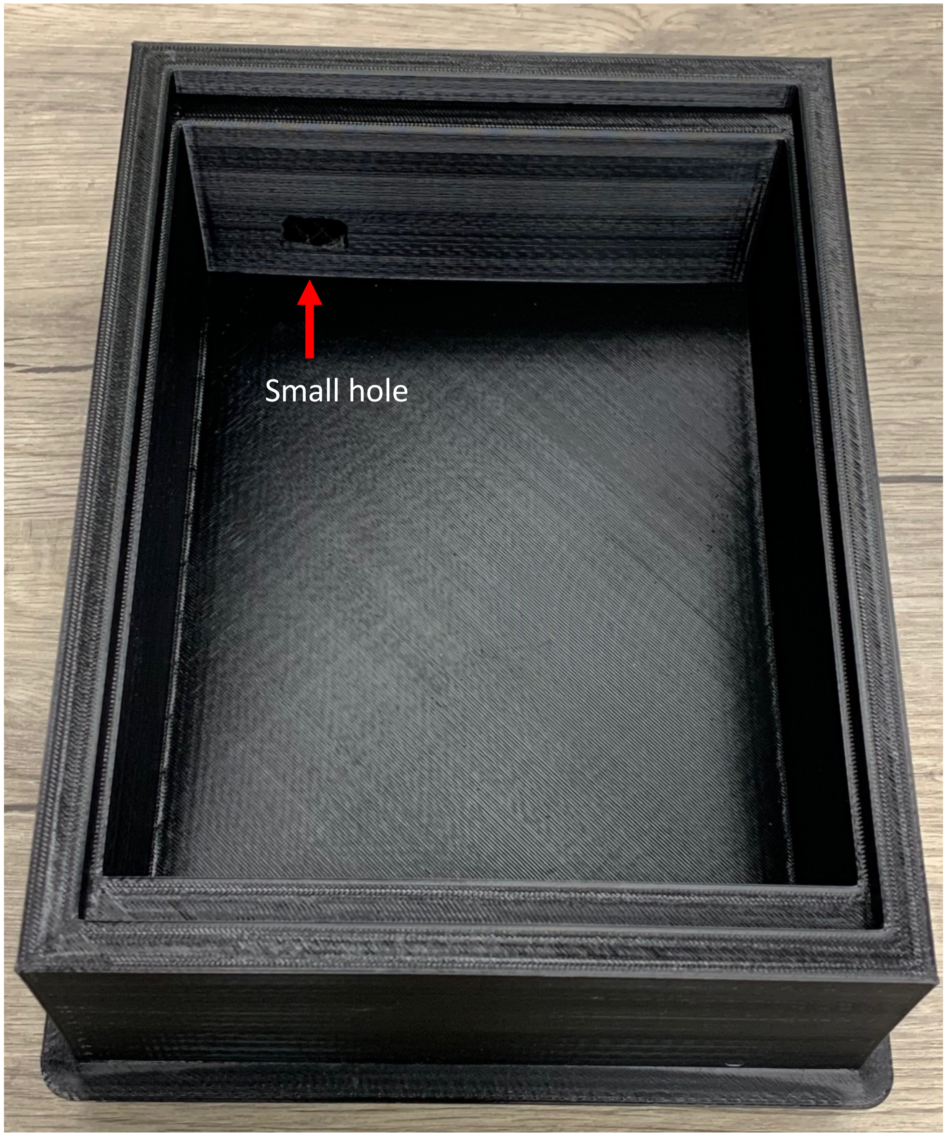

A small hole (red arrow) was drilled into the base to allow the USB cable for the DAQ board to pass. The photodiode mount fits / slides into the top of the base.

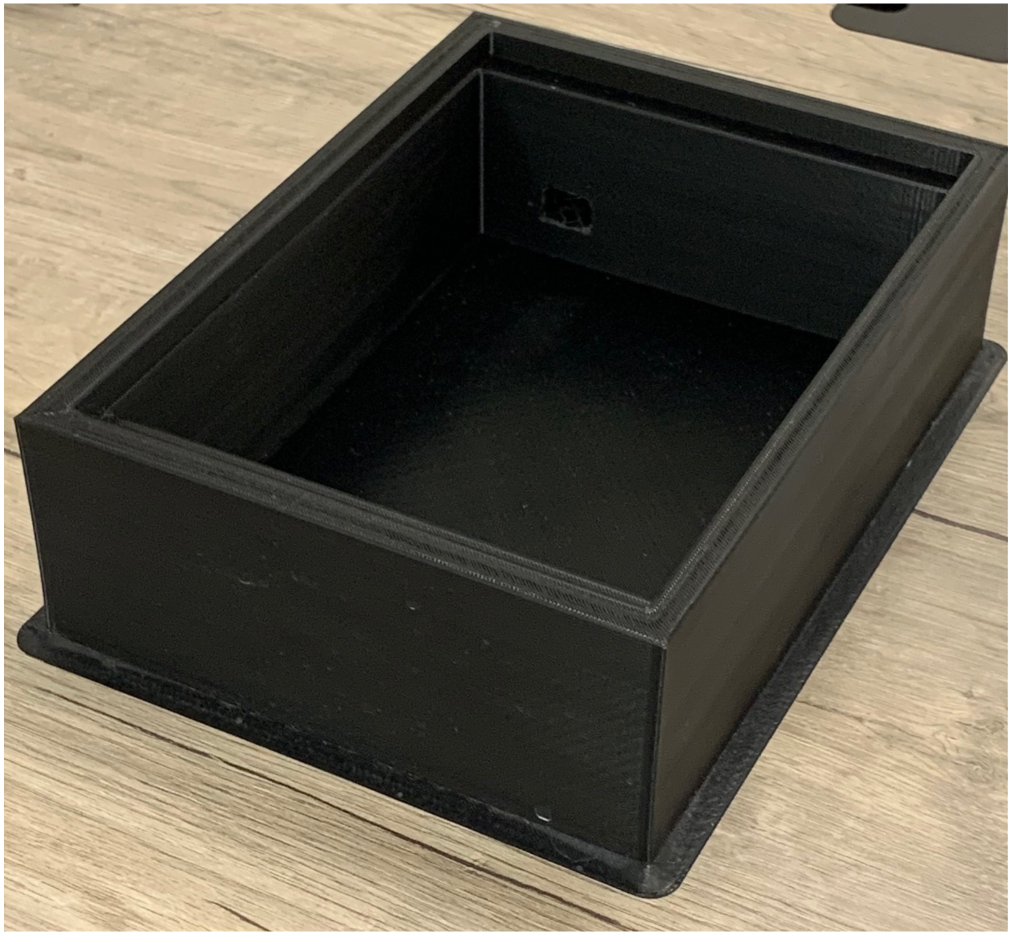

Collimation Screen (collimation screen.stl)

The collimation screen is placed over the well plate during operation. Its purpose is to reduce cross-talk between channels and stray light. To help assure consistent path lengths for absorption measurements.

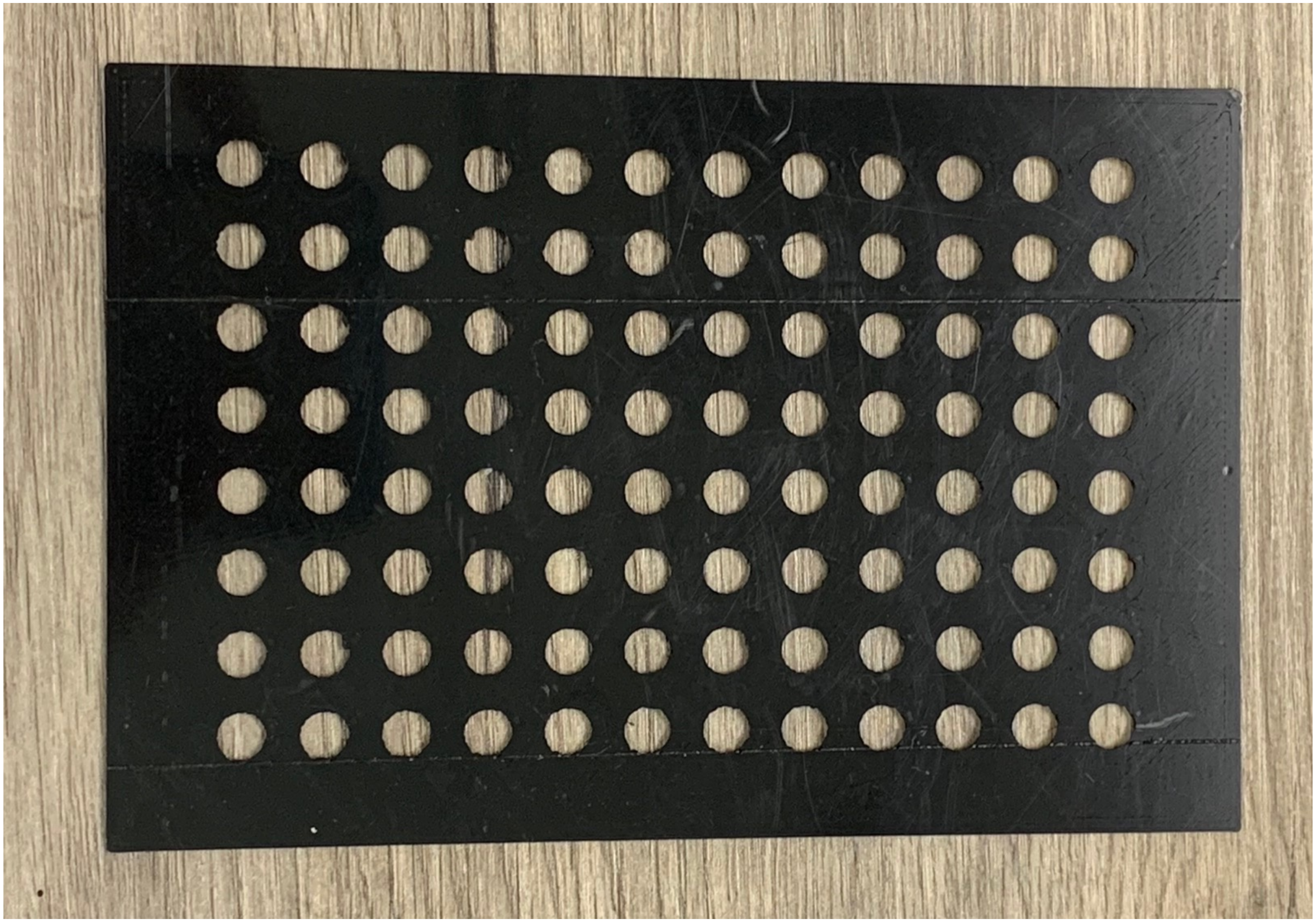

Assembled Apparatus

(A) Assembled plate reader on benchtop

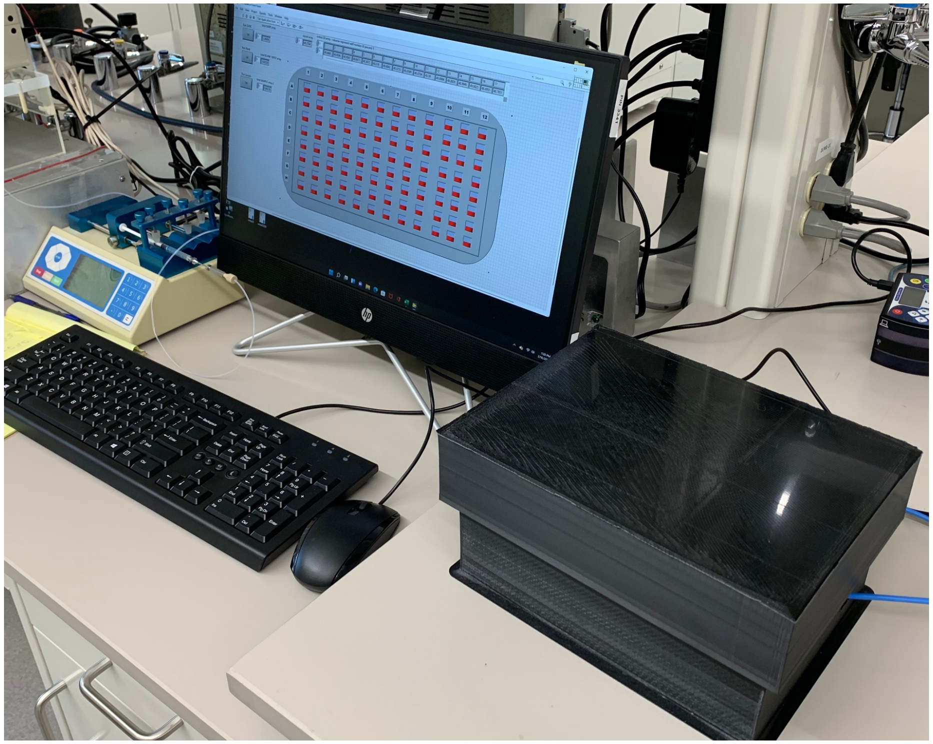

(B) Plate reader with top removed

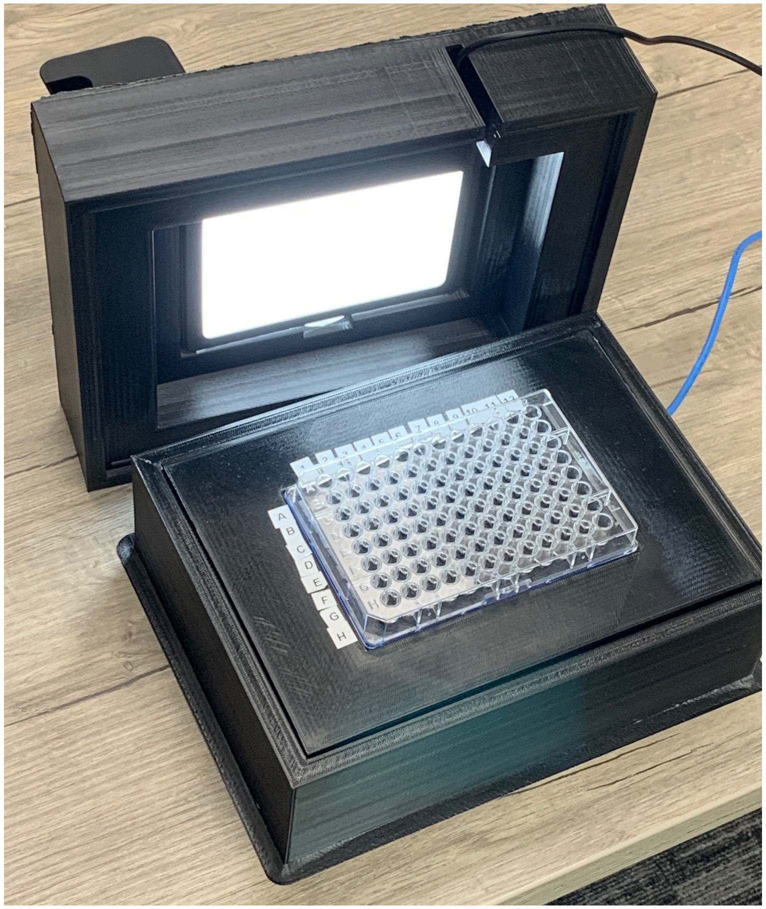

Software Interface

LabView program is designed to run continuously. When user clicks on button on left, program carries out certain function. Dark signals and 100% T signals must be acquired prior to collecting analytical data.

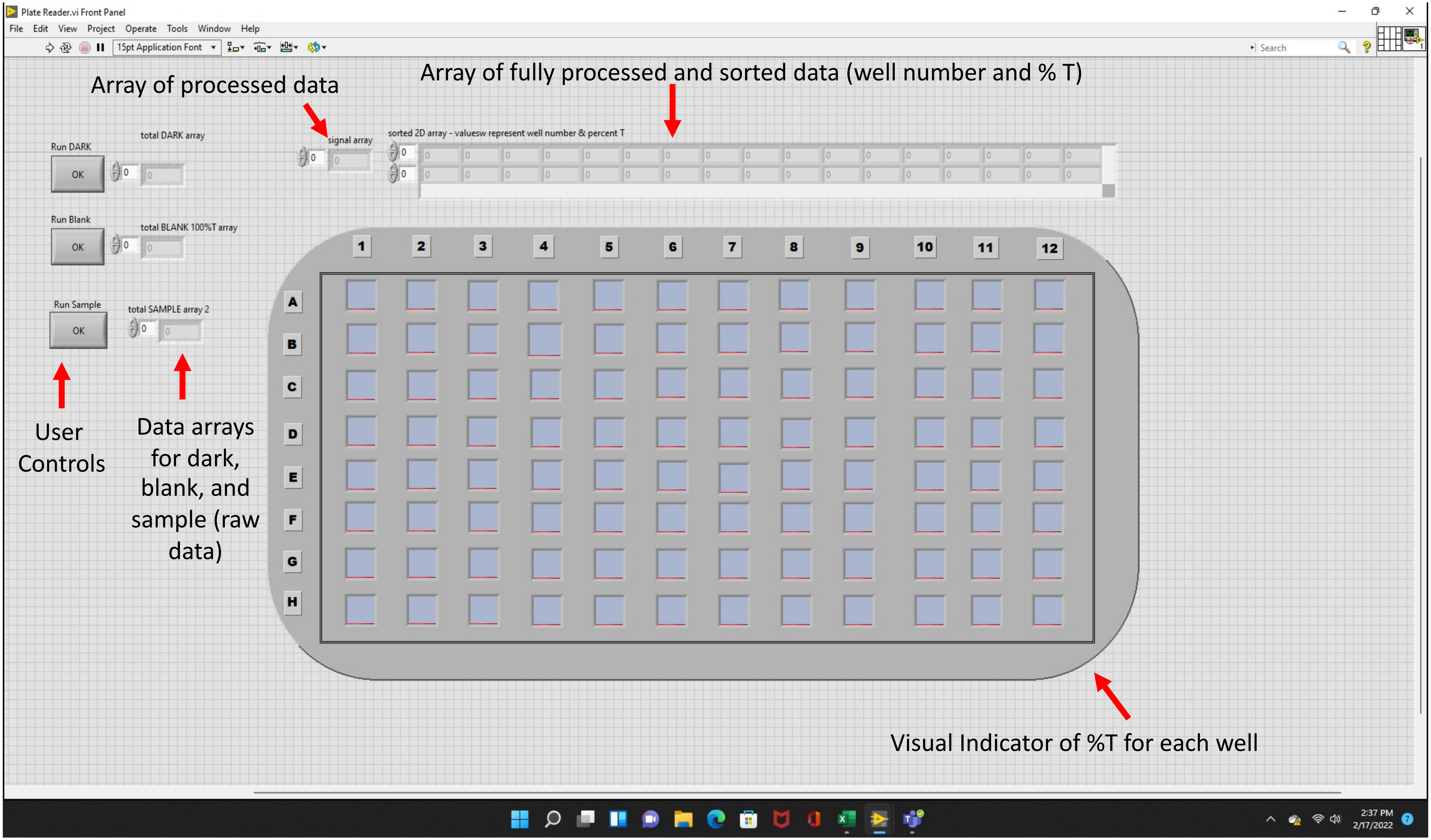

Supplement: Supplementary file 1 [file sensors-22-03242-s001.zip › sensors-1634802-supplementary.pdf]
